# Plant growth-promoting rhizobacteria *Burkholderia vietnamiensis* B418 inhibits root-knot nematode on watermelon by modifying the rhizosphere microbial community

**DOI:** 10.1038/s41598-022-12472-2

**Published:** 2022-05-19

**Authors:** Minmin Liu, Joshua Philp, Yilian Wang, Jindong Hu, Yanli Wei, Jishun Li, Maarten Ryder, Ruey Toh, Yi Zhou, Matthew D. Denton, Yuanzheng Wu, Hetong Yang

**Affiliations:** 1 School of Bioengineering, Qilu University of Technology (Shandong Academy of Sciences), Jinan, 250353 China; 2 Shandong Provincial Key Laboratory of Applied Microbiology, Ecology Institute, Qilu University of Technology (Shandong Academy of Sciences), Jinan, 250103 China; 3 China-Australia Joint Laboratory for Soil Ecological Health and Remediation, Ecology Institute, Qilu University of Technology (Shandong Academy of Sciences), Jinan, 250103 China; 4 School of Agriculture, Food and Wine, The University of Adelaide, Urrbrae, 5064 Australia

**Keywords:** Environmental microbiology, Microbial ecology

## Abstract

*Burkholderia vietnamiensis* B418 is a multifunctional plant growth-promoting rhizobacteria (PGPR) strain with nitrogen-fixing and phosphate-solubilizing capability which can be employed for root-knot nematode (RKN) management on various crops and vegetables. Here we investigated the control efficacy of *B. vietnamiensis* B418 inoculation against RKN on watermelon, applied either alone or combined with nematicides fosthiazate or avermectin, and their effects on bacterial and fungal microbiomes in rhizosphere soil. The results of field experiments showed individual application of B418 displayed the highest control efficacy against RKN by 71.15%. The combinations with fosthiazate and avermectin exhibited slight incompatibility with lower inhibitory effects of 62.71% and 67.87%, respectively, which were still notably higher than these nematicides applied separately. Analysis of microbiome assemblages revealed B418 inoculation resulted in a slight reduction for bacterial community and a significant increment for fungal community, suggesting that B418 could compete with other bacteria and stimulate fungal diversity in rhizosphere. The relative abundance of Xanthomonadales, Gemmatimonadales and Sphingomonadales increased while that of Actinomycetales reduced with B418 inoculation. The predominate Sordariomycetes of fungal community decreased dramatically in control treatment with B418 inoculation whereas there were increments in fosthiazate and avermectin treatments. Additionally, nitrogen (N) cycling by soil microbes was estimated by quantifying the abundance of microbial functional genes involved in N-transformation processes as B418 has the capability of N-fixation. The copy number of N-fixing gene *nifH* increased with B418 inoculation, and the highest increment reached 35.66% in control treatment. Our results demonstrate that *B. vietnamiensis* B418 is an effective biological nematicide for nematode management, which acts through the modulation of rhizosphere microbial community.

## Introduction

Root-knot nematodes (RKN), *Meloidogyne* spp., are highly polyphagous sedentary parasites capable of infesting a wide range of crops especially in greenhouse vegetable cultivation^1,2^. During infestation, RKN can incite obvious knots or galls on plant roots, destroy the normal structure of the roots, compete with the host for water and nutrition, and make the host susceptible to secondary pathogens^3–5^. Globally RKN damage is estimated to cause an annual economic loss of over $100 billion, accounting for about 12.6% of total crop losses^6^.

The control of RKN in intensive vegetable cultivation systems relies heavily on fumigants, carbamate, and organophosphate nematicides^7^. Currently there are three non-fumigant nematicides available in China: fosthiazate, avermectin, and fluopyram^8^. Fosthiazate is an organophosphorus acetylcholinesterase inhibitor that adversely disrupts the function of nervous system in nematode synapses and subsequently reduces the extent of root invasion^9^. Avermectin is a natural macrocyclic polyketide initially produced by *Streptomyces avermitilis*, which can open glutamate-gated chloride channels and paralyze nematodes specifically and irreversibly^10,11^. Yet the extensive reliance on fumigants and chemical nematicides is arousing severe concerns regarding environmental and human health issues^12^.

The use of biocontrol agents to reduce RKN damagehas provided an environment-friendly alternative^13^. Plant growth-promoting rhizobacteria (PGPR) are beneficial bacteria that colonize the rhizosphere and plant roots resulting in enhancement of plant growth or protection against plant pathogens via production and secretion of various regulatory chemicals^14^. PGPR-based bioagents such as the strains of genus *Bacillus* spp., *Burkholderia* spp., and *Pseudomonas* spp. have been assessed for the control of RKN, which can attack and kill RKN by diverse processes including capturing, parasitizing, and producing toxins and enzymes, and induce plant systemic acquired resistance^15,16^. Luo et al. reported that *Bacillus mycoides* R2 exhibited control efficiency by 90.94% against *M. incognita* in a pot experiment and the nematicidal compound was isolated and identified as styrene^17^. Siddiqui and Shaukat discovered that *Pseudomonas fluorescens* CHA0 induced systemic resistance against RKN via a signal transduction pathway which was independent of salicylic acid accumulation in tomato roots^18^. Li et al. found *Burkholderia ambifaria* BC-F released a diffusable metabolite which inhibited egg hatch and mobility of second-stage juveniles (J2) of *M. incognita*^19^. Khanna et al. used *P. aeruginosa* and *Burkholderia gladiol* to reduce the negative effect of RKN in seedlings and improved the growth effect and antioxidative potential of tomato^20^. Although many microorganisms have been isolated and selected as bioagents for controlling RKN, the control effect of these agents are often unstable in field^21^. Nevertheless, worldwide researches suggest that sustainable nematode-control methods are available and preferred using integrated measures^22,23^.

The interactions among RKN, biocontrol agents, and rhizosphere microbiota have not been fully illustrated in the soil ecosystem^5,24^. There is still limited information on the influence of the inoculation of biocontrol agents on microbial communities and activities present in the rhizosphere. As a critical role for plant health, rhizosphere microbiota is related to their contribution to plant nutrition and biomass^25^. Microbial communities supply plant-available nitrogen (N) through biological N fixation and mineralization, and convert N into multiple chemical forms by nitrification and denitrification processes^26,27^. Babic et al. reported an increased alfalfa yield with seed inoculation by two *Sinorhizobium meliloti* strains, which was related to enhanced nitrogen content and the abundances of N-fixing gene (*nifH*) and ammonia-oxidizing gene (*amoA*)^28^. Ke et al. also found improved maize growth and nitrogen content inoculated by endophytic *P. stutzeri* A1501 that could be attributed to the positive effect of A1501 on the population of N-cycling communities and functional genes transcripts of *nifH* and *amoA*^29^. With the development of metagenomics and metabolomics analyses, the study of microbial ecosystem structure can be performed to a greater depth and accuracy^30^.

*Burkholderia vietnamiensis* B418, belonging in the *B. cepacia* complex, is a multifunctional PGPR strain with nitrogen-fixing and phosphate-solubilizing capability which can be described within the assembly of the B418 genome^31^. We have identified several nematode virulence factors from B418 such as degrading enzymes of chitinase and protease, and secondary metabolites including cyclic dipeptide and siderophore, which exhibited high inhibitory effect against RKN on cucumber and eggplant^32–34^. In this study, we examined the nematode control efficacy and corresponding influences on the microbiome assemblages and abundances of N-cycling genes in the rhizosphere of watermelon under continuous cropping greenhouse conditions, with B418 inoculated either alone or in combination with nematicides fosthiazate or avermectin.

## Results

### Control efficacy of B418 inoculation against RKN

The effects of different treatments on the reduction rate of nematode density and control efficacy against RKN are presented in Table 1. All treatments reduced nematode density to some extent compared with the negative control (CK−). The highest reduction rate and control efficacy was observed with *B. vietnamiensis* B418 inoculation alone by 74.84% and 71.15%, respectively. The introduction of B418 enhanced the inhibitory effects of chemical nematicide fosthiazate (from 38.92% to 62.71%) and biological nematicide avermectin (from 59.24% to 67.87%), which were still lower than that of B418 inoculated alone, indicating there was slight incompatibility within the combinations of B418 with fosthiazate and avermectin.

**Table 1:** Effects of different treatments on the density of root knot nematode juveniles in rhizpsphere soils of greenhouse-grown watermelon. Data in the table are mean of 3 replicates. Means followed by different letters within a column are significantly different (*P* < 0.05, Duncan’s test). CK: control; FOST: fosthiazate; AVM: avermectin. (+ /-) stands for with and without B418 inoculation.

Treatments	Nematode density (/100 g soil)	Reduction rate of nematode density (%)	Disease severity (%)	Control efficacy (%)
CK ( −)	624.33 ± 28.92 a	–	86.18 ± 7.27 a	–
CK ( +)	158.67 ± 13.58 c	74.84	24.86 ± 4.21 b	71.15
FOST ( −)	367.00 ± 21.52 a	41.03	52.64 ± 5.42 a	38.92
FOST ( +)	205.33 ± 14.57 c	67.15	32.14 ± 6.36 c	62.71
AVM ( −)	245.00 ± 21.66 b	61.22	35.13 ± 5.81 b	59.24
AVM ( +)	176.67 ± 20.50 c	71.70	27.69 ± 4.73 b	67.87

### The effect of B418 inoculation on microbial OTU composition

**Figure 1:**
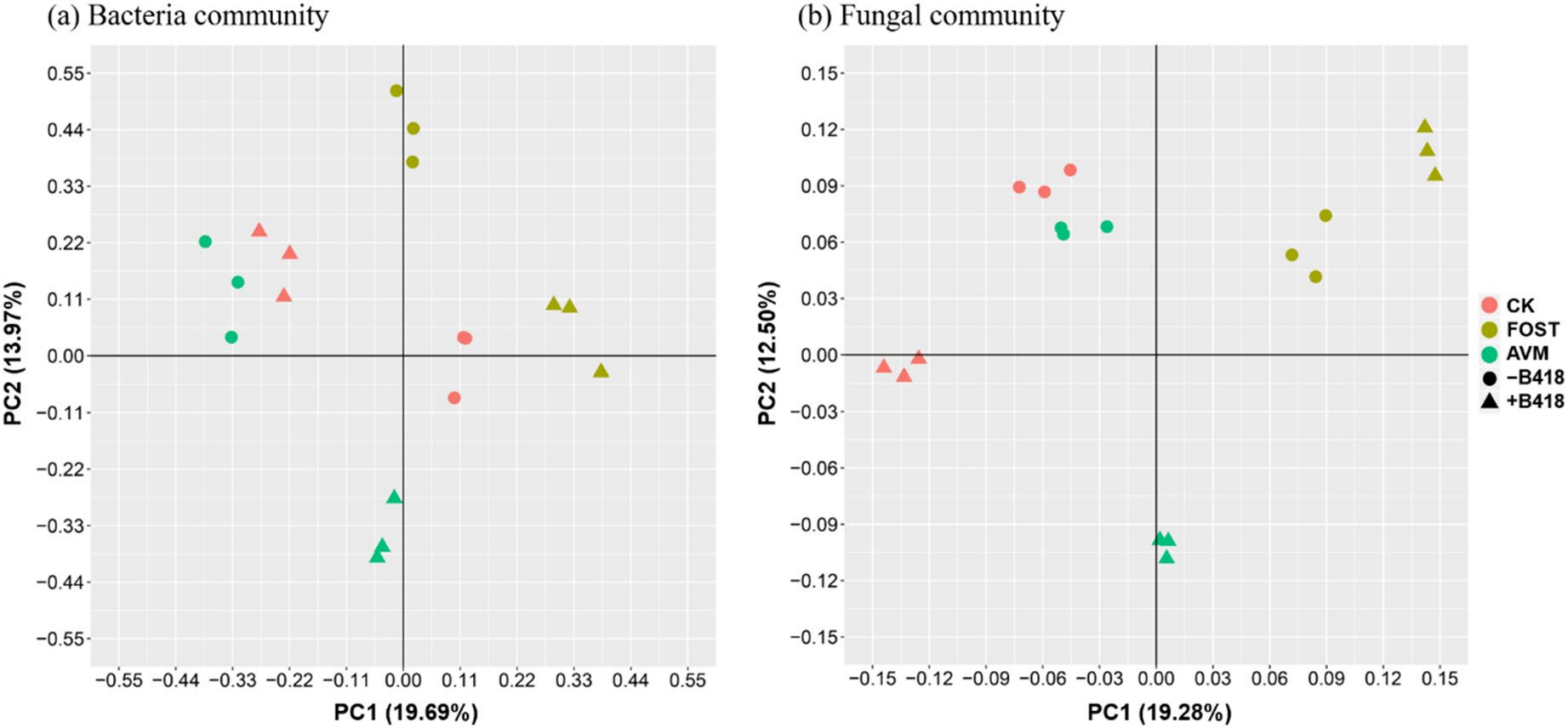
The effects of *Burkholderia vietnamiensis* B418 inoculation on microbial composition of rhizosphere soil of watermelon. Biplots included principal component analysis (PCA) with weighted UniFrac distances for (**a**) bacterial community and (**b**) fungal community in different treatments. CK: control; FOST: fosthiazate; AVM: avermectin. (+ /−) stands for with and without B418 inoculation.

To evaluate the similarity and difference of the microbial community structures in all treatments, a principal component analysis (PCA) based on weighted UniFrac distance metrics was performed. As delineated in Fig. 1, PC1 and PC2 accounted for 19.69% and 13.97% of the total variation for bacterial community, and PC1 and PC2 represented 19.28% and 12.50% of the variance for fungal community, respectively. The results indicated that treatments with and without B418 inoculation showed clear separation for both bacterial and fungal communities by PCA analysis, with significant effect on the composition of bacterial community and moderate one of fungal community.

The constrained principal component analysis identified the effect of strain B418 treatment and its interaction with other disease control treatments (Table 2). The application of B418 increased bacterial 16S rRNA sequences from 9.1% to 34.6%, and fungal ITS-2 rRNA sequences from 7.1% to 30.3%, which both exhibited more variation than the disease control treatments.

**Table 2:** *Burkholderia vietnamiensis* B418 and its interaction with other disease control treatments.

Treatments	Bacterial 16S rRNA sequences		Fugal ITS-2 rRNA sequences	
	R^2^	*P*-value	R^2^	*P*-value
Disease control treatment	9.1%	0.001	7.1%	0.001
*B. vietnamiensis* B418	33.3%	0.001	30.6%	0.001
Disease control treatment*B418	34.6%	0.001	30.3%	0.001

**Figure 2:**
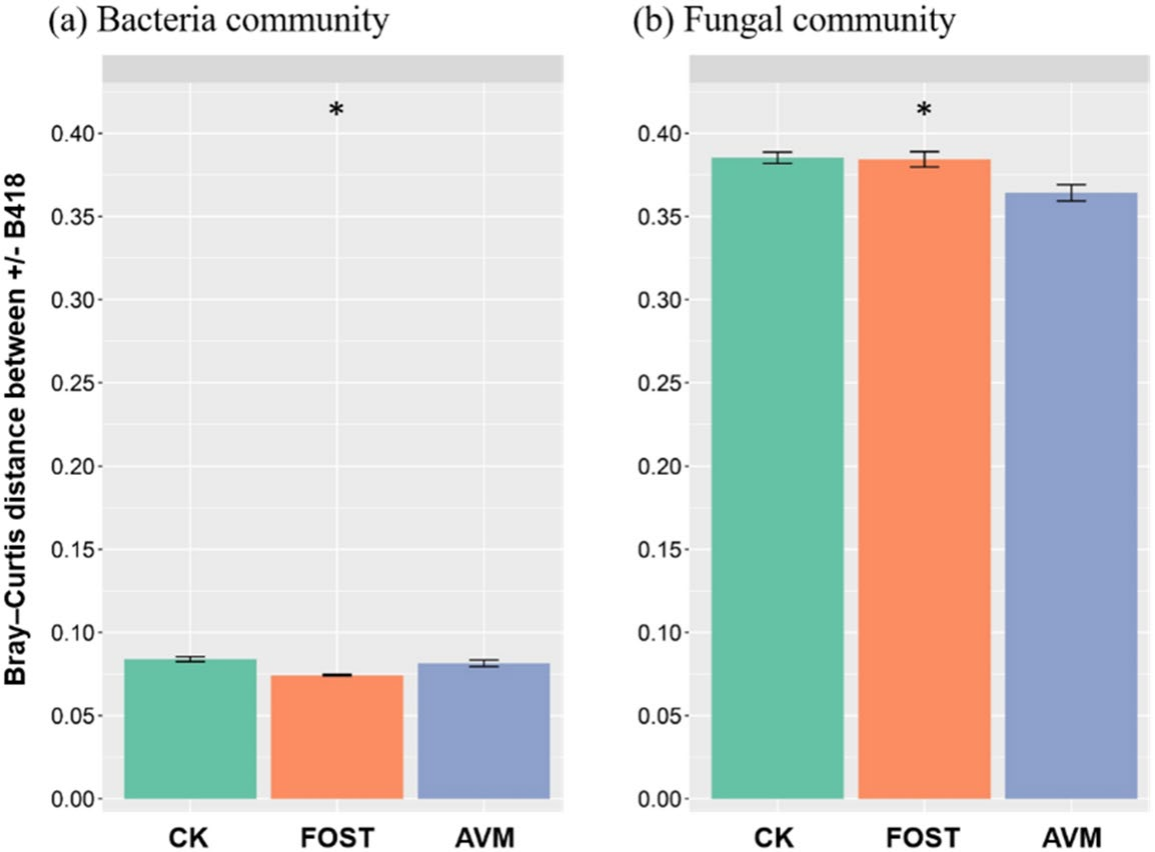
The relative abundance by Bray–Curtis distance of (**a**) bacterial community and (**b**) fungal community in the rhizosphere of different treatments with *Burkholderia vietnamiensis* B418 inoculation. Error bar indicates standard error at *P* = 0.05 in the ANOVA test for microbial content. CK: control; FOST: fosthiazate; AVM: avermectin.

Relative abundances, analyzed by Bray–Curtis distance, were presented in Fig. 2. The effect of B418 inoculation was significant while the difference was small (*P* < 0.05). For the bacterial community, fosthiazate treatment reduced bacterial population density compared with control and avermectin treatments with B418 inoculation. For the fungal community, the lowest relative abundance was observed in avermectin treatment.

### The effect of B418 inoculation on OTU diversity

**Figure 3:**
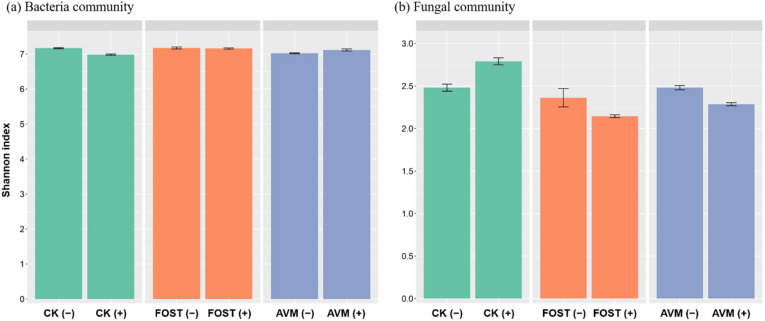
The effects of *Burkholderia vietnamiensis* B418 inoculation on microbial alpha diversity in the rhizosphere of greenhouse-grown watermelon. Columns show the Shannon index for (**a**) bacterial communities and (**b**) fungal communities within and among different treatments. CK: control; FOST: fosthiazate; AVM: avermectin. (+ /−) stands for with and without B418 inoculation.

The Shannon index was used to estimate the alpha diversity and the proportion of variation within and between treatments. The effects of factor 1 and factor 2 applications were significant for both the bacterial and fungal communities, and the variation in fungal diversity was larger than that in bacterial diversity (Fig. 3a,b). Interestingly, B418 inoculation caused a slight reduction for the bacterial community and a significant increment for the fungal community in all treatments except fosthiazate. For the bacterial community, B418 inoculation in control and fosthiazate treatments led to slight declines in diversity, while a slight increase was observed in avermectin treatment. Conversely, B418 inoculation resulted notable increased diversity for the fungal community in control treatment, while a distinct decline was observed in fosthiazate and avermectin treatments. The results suggested that B418 could compete with other bacteria and stimulate fungal diversity in the rhizosphere.

### The effect of B418 inoculation on microbial taxa

**Figure 4:**
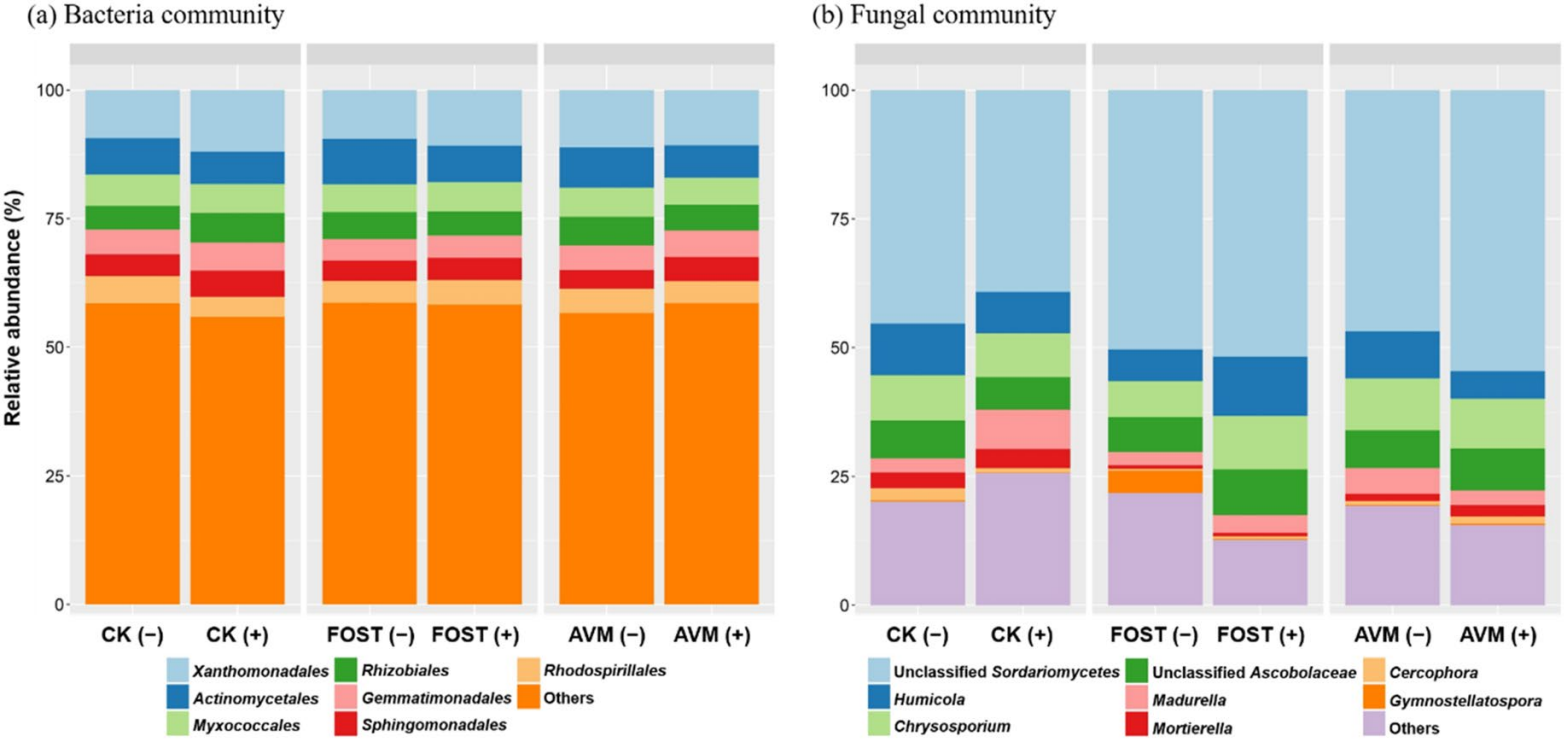
Taxonomic composition influenced by *Burkholderia vietnamiensis* B418 inoculation in different treatments at (**a**) order level for bacteria, and (**b**) genus level for fungi. CK: control; FOST: fosthiazate; AVM: avermectin. (+ /−) stands for with and without B418 inoculation.

The differences in predominate microbial taxa in the rhizosphere between treatments with and without B418 inoculation were shown in Fig. 4. The taxonomic level was set at Order for bacteria, and Genus for fungi, but in higher taxonomic groupings where genus could not be identified. Xanthomonadales, Actinomycetales, Myxococcales, Rhizobiales, Gemmatimonadales, Sphingomonadales, and Rhodospirillales were the predominate orders in the rhizosphere bacterial community of all treatments (Fig. 4a). B418 inoculation resulted in a higher abundance of Xanthomonadales, Gemmatimonadales and Sphingomonadales, and lower proportions of Actinomycetales in all treatments (Fig. 4a). A higher abundance of Myxococcales and lower proportions of Rhizobiales were also found in fosthiazate and avermectin treatments with B418 inoculation.

Unclassified Sordariomycetes were predominate in the rhizosphere fungal communities, and there was a high percentage of *Humicola*, *Chrysosporium* and unclassified Ascobolaceae in all treatments (Fig. 4b). Sordariomycetes decreased dramatically in control treatment with B418 inoculation whereas there were increments observed in fosthiazate and avermectin treatments. For control treatment, *Humicola*, *Chrysosporium*, Ascobolaceae, and *Cercophora* all decreased with B418 inoculation whereas *Madurella* and *Mortierella* increased. For fosthiazate treatment, B418 inoculation caused an increase in Sordariomycetes, *Humicola*, *Chrysosporium*, and Ascobolaceae together with a significant decrease in *Gymnostellatospora*. And for avermectin treatment, a clear increase of Sordariomycetes and decrease of *Humicola* and *Madurella* were observed with B418 inoculation.

### The effect of B418 inoculation on the abundance of *Burkholderia* spp.

**Figure 5:**
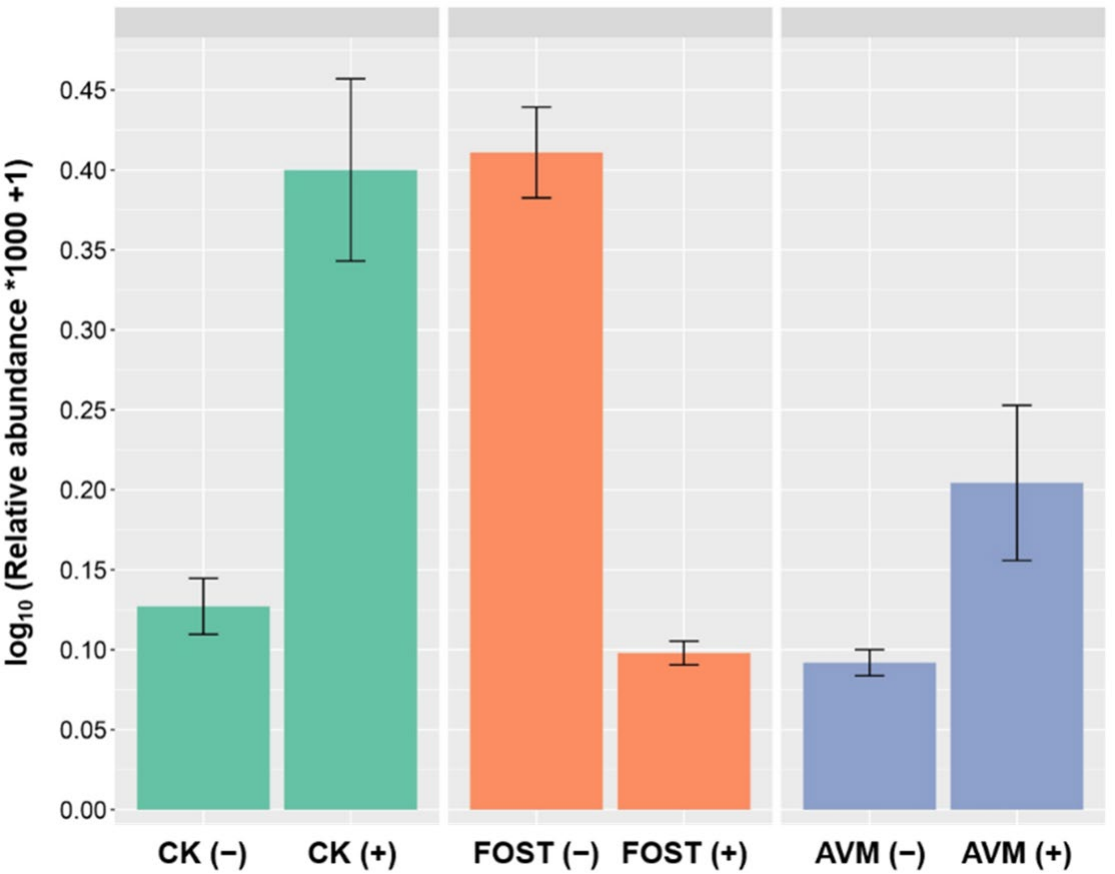
The relative abundance of *Burkholderia* spp. in different treatments. Error bar and * indicate standard error at *P* = 0.05 in the ANOVA test for microbial content. CK: control; FOST: fosthiazate; AVM: avermectin. (+ /−) stands for with and without B418 inoculation.

The relative abundance of *Burkholderia* spp. in all treatments were monitored by detection of viable cells (colony forming units, CFU) using their OTUs (Fig. 5). There were significant increments observed for both control and avermectin treatments with B418 inoculation, which increased around 3.1-fold for control treatment. On the contrary the relative abundance of *Burkholderia* spp. declined sharply in fosthiazate treatment with B418 inoculation.

### The effect of B418 inoculation on the abundance of N-cycling genes

**Figure 6:**
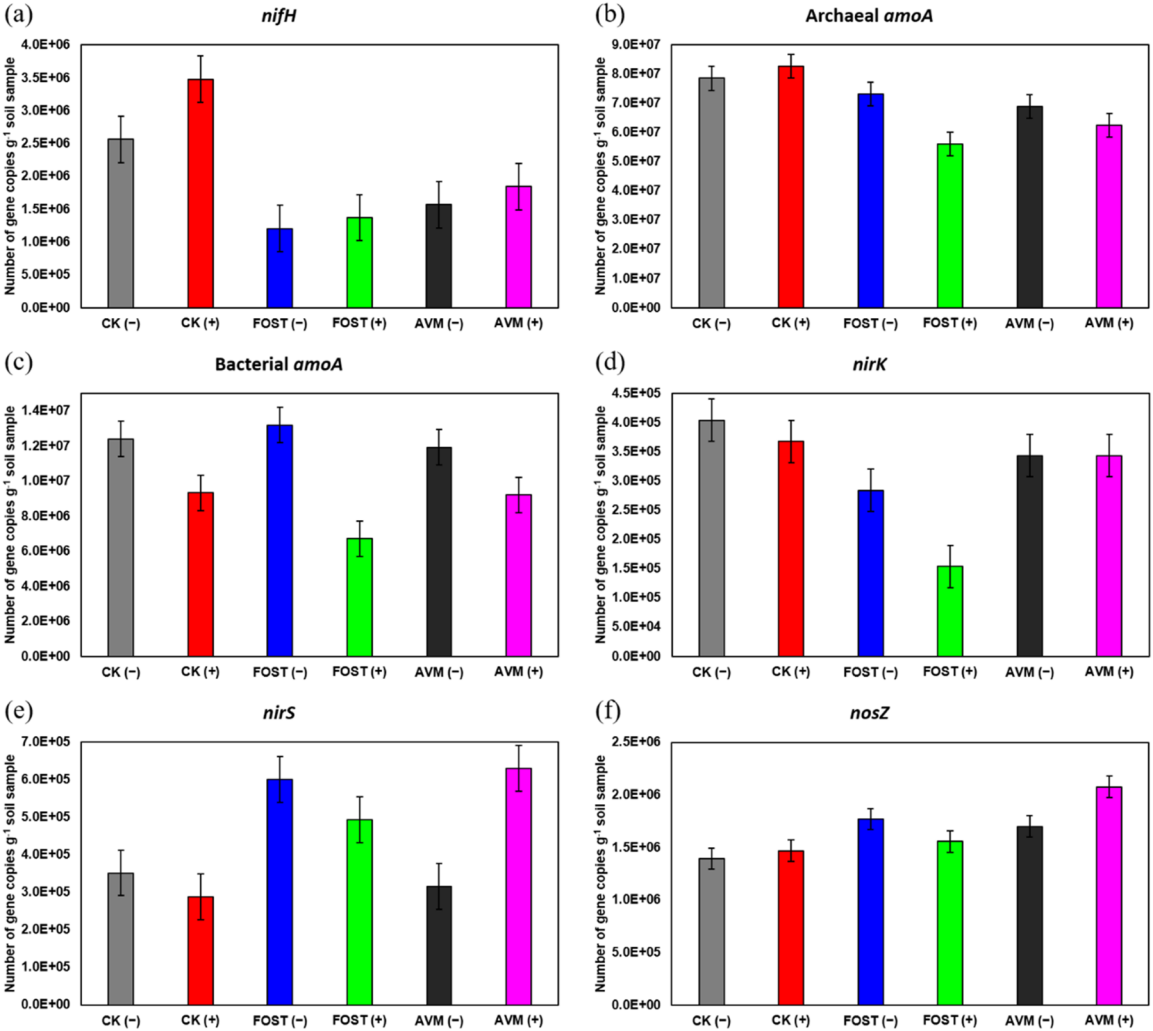
Quantitative PCR of nitrogen-cycling gene abundances. The abundance of six N-cycling genes involved in nitrogen fixation (a, *nifH*), ammonia oxidation by archaea (b, archaeal *amoA*) and bacteria (c, bacterial *amoA*), and denitrification (d-f, *nirK*, *nirS*, *nosZ*) in different treatments. CK: control; FOST: fosthiazate; AVM: avermectin. (+ /−) stands for with and without B418 inoculation. Error bar indicates standard error at *P* = 0.05 in the ANOVA test for microbial content.

Five functional genes related to nitrogen fixation (*nifH*), nitrification (archaeal *amoA* and bacterial *amoA*), and denitrification (*nirK*, *nirS*, and *nosZ*) were included for the estimation of N cycling by soil microbes since B418 has been verified for the capability of N fixation. As delineated in Fig. 6, the abundance of N-fixing *nifH* gene was increased in all treatments with B418 inoculation. The highest increment reached 35.66% in control treatment (from 2.56 × 10^5^ to 3.48 × 10^5^) which was 2~3-fold greater than fosthiazate and avermectin treatments (1.20~1.37 × 10^5^ and 1.57~1.84 × 10^5^, respectively). B418 inoculation resulted in decrement for bacterial *amoA*, *nirK*, and *nirS* in control treatment, and slight increment for archaeal *amoA* and *nosZ*. There was decrement observed for archaeal *amoA*, bacterial *amoA*, and *nirK* in avermectin treatment, and increment for *nirS* and *nosZ* with B418 inoculation. Interestingly, all nitrification and denitrification genes decreased dramatically in fosthiazate treatment.

## Discussion

The results from field experiments in this study showed that *B. vietnamiensis* B418 inoculation could significantly reduce the incidence of RKN by 71.15% on watermelon, while potential conflicts were detected when combined with nematicides fosthiazate and avermectin. Although the control efficacy of B418 with fosthiazate or avermectin were lower than the individual application of B418, these combinations could reduce the utilization of nematicides by a synergistic additive effect on nematode suppression. There were several studies suggesting the combination of biocontrol agents with fosthiazate or avermectin for nematode control and plant promotion^35^. Combined application of *Syncephalastrum racemosum* and avermectin reduced the number of nematode galls and enhanced cucumber growth^36^. The reduction of *M. incognita* and the increment of tomato yield were observed using avermectin together with *P. fluorescens* or *Trichoderma viride*^37,38^. However, in contrast to previous reports, the suppression efficacy of both combinations with fosthiazate and avermectin was still lower than B418 inoculated alone. This might be attributed to the incompatibility of B418 with both nematicides, especially with fosthiazate the inoculation of B418 even resulted in dramatic decline of *Burkholderia* spp. and reduced bacterial population density.

As PGPR are considered as opportunist and can rapidly adapt to the environment according to variations in the nature and quantity of root exudates, they are capable of colonizing the rhizosphere and establishing a tight relationship with roots which are usually favored by plant host^39,40^. The beneficial effects of PGPR on plant growth involve either direct mechanism such as biofertilization (facilitation of nutrient uptake including nitrogen and phosphorus primarily) and phytostimulation (production of plant growth promoting hormones), or indirect mechanism as biocontrol agents that antagonize the deleterious effects of phytopathogens by producing inhibitory substances or by inducing plant systemic resistance^41^. Our previous researches have verified the capability of B418 as PGPR strain both with direct enhancement of plant growth by nitrogen fixation and phosphate solubilization and with indirect promotion by degrading enzymes and secondary metabolites^32–34^. B418 inoculation on watermelon exhibited symbiotic and/or mutualistic interactions with microbial community in the rhizosphere, presenting notable change on the composition of the bacterial community and marginal difference on fungal community as indicated by PCA analysis. Ciccillo et al. reported *Burkholderia ambifaria* MCI 7 promoted maize growth significantly and brought about an abrupt decrease in bacterial diversity when applied as a seed treatment^42^. Jo et al. discovered there was also an obvious change in soil bacterial community structure due to the application of *Bacillus thuringiensis*^43^. Similarly, Wang et al. discussed the significant effect of two microbial co-inoculants with the same three strains (*Ensifer* sp., *Acinetobacter* sp., and *Flavobacterium* sp.) on soil bacterial communities that were strongly separated by the different microbial inoculation treatments^44^. These examples illustrate that the impacts of PGPR on rhizosphere indigenous bacterial communities are likely to be a net result of both positive and negative effects^45^.

The diversity of the rhizosphere microbial community structure is an important indicator reflecting the health status of soil^46^. From the microbiome results presented here it could be concluded that the effect of B418 inoculation was significant while the difference was small. This was possibly because all samples in this study were collected from the rhizosphere, unlike the previous papers where different compartments from across the soil and plant root interface were used^44,47^. Application of B418 resulted in a slight reduction for the bacterial community diversity (Shannon index) and an increase for the fungal community, except for the combination with avermectin. These opposite trends for the impact of B418 inoculation on bacterial and fungal community diversity demonstrated that strain B418 could compete with other bacteria in the rhizosphere and stimulate fungal diversity simultaneously^48^. The diversity of rhizosphere microorganisms in B418 treatment was not significantly different from that in control treatment, indicating that B418 inoculation had slight effects on the soil microbial diversity in the roots of watermelon.

The general predominance of Xanthomonadales, Rhizobiales, Sphingomonadales, Rhodospirillales, and Actinomycetales that we observed was expected, and the first four groups were all subdivisions of Proteobacteria. As predominate members of the rhizosphere microbial community, Proteobacteria and Actinobacteria were suggested to be dynamic taxa associated with plant disease suppression in previous studies using DNA metagenomics^49,50^. Zhou et al. found that the phyla Proteobacteria, Bacteroidetes, Acidobacteria, Actinobacteria, and Chloroflex were more abundant in non-infested microbiota than in *M. incognita-*infested microbiota^51^. Interestingly, B418 inoculation resulted in an increase in Xanthomonadales, Gemmatimonadales, and Sphingomonadales whereas Actinomycetales decreased in all treatments. This was consistent with the metagenomic sequencing results by Zou et al. that oxytetracycline treatment for the control of RKN enhanced the abundance of Proteobacteria phylum but decreased the abundance of Actinobacteria^52^. Kong et al. also reported the contrary trends of significantly lower relative abundance of Burkholderiales and higher relative abundance of Actinomycetales, which might be caused by the occupation of nutritional niches in the rhizosphere by Proteobacteria and Actinobacteria^53^.

Gemmatimonadales are a recently described bacterial group whose members are widespread in soil habitats yet mostly non-culturable, and they can accumulate polyphosphate and play important roles in phosphorus removal^54^. The increase in Gemmatimonadales with B418 inoculation might be attributed to the phosphate-solubilizing capability of B418 causing the accumulation of phosphorus in the rhizosphere. Myxococcales most commonly inhabit topsoil, especially soil that is rich in organic matter^55^. Schmidt et al. identified three members of the order Myxococcales (identified as the genera *Phaselicystis*, *Archangium*, and *Myxococcus*) and two members of the order Burkholderiales (identified as the genera *Rhizobacter* and *Achromobacter*) were indicators of organic environments^56^. The increase in Myxococcales might be associated with the application of B418 for its multiple PGPR functions in soil, which was in line with previous studies revealing these orders to be organic-system-specific.

Compared with the bacterial community of the watermelon rhizosphere, the fungal community was more diverse and variant with B418 inoculation. Unclassified Sordariomycetes were predominate of fungal community, which were dramatically decreased in control treatment with B418 inoculation. Hu et al characterized that Ascomycota was predominate in the fungal communities associated with soybean cyst nematode, comprising more than half of the OTUs at each sampling time point^57^. Sordariomycetes were one of the highest number of OTUs at the class level in Ascomycota and particularly showed significantly higher relative abundance in longer soybean crop sequences and continuous monoculture^57^. Other most abundant genera were *Humicola*, *Chrysosporium*, *Mortierella*, *Madurella*, *Cercophora*, and *Gymnostellatospora*. However, there were no obvious patterns that all treatments followed after B418 inoculation. *Humicola*, *Chrysosporium*, Ascobolaceae, and *Cercophora* all decreased with B418 inoculation whereas *Madurella* and *Mortierella* increased in control treatment. *Humicola* could produce cellulose acetate deacetylase with biodegradative activity in disposal environments and result in an increase in the levels of lignin, cellulose and hemicellulose in the soil, which generally lead to an increase in the number of nematodes that feed on crop plants^58^. The decrease of *Humicola* with B418 inoculation was favorable for the control of RKN. On the contrary, *Mortierella* are known to decompose chitin and have the ability to suppress many pathogens^59^. For example, *M. alpina* can synthesize alkaloid antibiotics and enhance the protection of plants against worms and phytopathogens^60^. Furthermore, both *M. alpina* and *M. signyensis* are apt at killing insect pests by inoculation or injection^61^. The increment of *Mortierella* with B418 application was in accordance with the control effect against RKN. These variations in fungal community indicated that B418 inoculation resulted in the inhibition of pathogen-related species and the enhancement of plant-beneficial ones.

It has been documented that *B. vietnamiensis* B418 is a nitrogen-fixing bacterium^31^, so the other N-fixing bacteria Rhizobiales were reduced in rhizosphere with B418 inoculation which was predictable and might be explained by the substitution of strain B418 that could supply N to plants with its ability to fix atmospheric nitrogen. With regard to nutrient uptake mechanisms, B418 was found to enhance the copy number of N-fixing gene *nifH* in all treatments. Biological N-fixation can transform nitrogen to the biologically usable ammonia, which is also generated through soil mineralization^62^. This process is essential to catalyze N-fixation, and the *nifH* gene is often used as a marker gene for the molecular analysis of N-fixing microbes^63^. The enhancement of *nifH* in this study was in consistency with the results of Pham et al. in which biological N-fixation by inoculated *Pseudomonas stutzeri* strain A15 was responsible for a small contribution to rice plant nitrogen^64^. However, other studies indicated that wild-type *P. stutzeri* A1501 and *nifH*-mutant colonized maize roots equally, and the deficiency of *nifH* gene does not affect the ability of N-fixing bacteria to colonize the maize plant^29^. Interestingly, Ouyang et al. reported that N fertilization significantly suppressed the *nifH* abundance in the rhizosphere, while N fertilization had no effect on the *nifH* abundance in the bulk soil^65^. This suggested that N-fixers were more sensitive to N addition in the rhizosphere and the application of N fertilization might reduce the potential of biological N input from free-living N-fixation. Dynarski and Houlton also reported that free-living N-fixation rate was significantly suppressed by N fertilization in natural terrestrial ecosystems^66^.

There were no consistent patterns found regarding N-cycling genes involved in nitrification (archaeal *amoA* and bacterial *amoA*) and denitrification (*nirS*, *nirK*, and *nosZ*). These genes were significantly correlated with each other, largely because a similar N fertilization effect was observed for these genes^67^. Ouyang et al. reported that N fertilization significantly increased archaeal *amoA*, bacterial *amoA*, *nirK*, *nirS*, and *nosZ*, indicating N fertilization stimulated the growth of nitrifiers and denitirifers in the rhizosphere relative to the bulk soil^65^.

In summary, the application of *B. vietnamiensis* B418 was able to reduce the incidence of root-knot nematode on watermelon significantly, and to reduce the utilization of other nematicides, while potential conflicts were detected when combined with fosthiazate and avermectin. The investigation of rhizosphere microbiome assemblages demonstrated that strain B418 was capable of competing with other bacteria in the rhizosphere and stimulating fungal diversity. As a member of the Betaproteobacteria, B418 inoculation resulted in a higher relative abundance of other Proteobacteria and lower relative abundance of Actinomycetales. The predominate Sordariomycetes decreased dramatically for fungal community with B418 inoculated alone whereas these population increased in the combination treatments. In addition, the abundance of N-fixing gene *nifH* increased in all treatments with B418 inoculation as B418 has been verified for the capability of N-fixation. Our results indicated that strain B418 could play a larger role in agricultural systems as a promising PGPR and biocontrol agent in future.

## Methods

### Microorganisms and plant species

*Burkholderia vietnamiensis* B418 (China General Microbiological Culture Collection Center, CGMCC No.1212) was isolated from barley soil and maintained at –80 °C as previously described^32^. Watermelon (*Citrullus lanatus*) cv. Jingxin used in this study was the same as previously planted in the greenhouse, provided by the Institute of Vegetables and Flowers of Shandong Academy of Agricultural Sciences. The study was performed in accordance with relevant local and national guidelines.

### Experimental location

This study was conducted in the greenhouse with a history of watermelon cultivation in Linqu County, Weifang City, Shandong Province, China (36°45′ N, 118°47′ E). A high incidence of RKN had been observed for over a decade in this greenhouse due to continuous cropping.

### Experimental design

The experiment was designed using a randomized design with 2 factors defining 6 treatments, and three replicates per treatment. The first factor was *B. vietnamiensis* B418 inoculation (i.e. inoculated, designated + hereafter; and uninoculated, designated − hereafter). The second factor was the application of chemical nematicide fosthiazate (FOST), biological nematicide avermectin (AVM), and control (CK). These two factors were combined to define six treatments listed in Table 3.

**Table 3:** Experimental treatments. Factor 1: ( +) with *B. vietnamiensis* B418 inoculation; ( −) without *B. vietnamiensis* B418 inoculation. Factor 2: Control (CK); Fosthiazate (FOST); Avermectin (AVM).

No	Treatments	Explanations
1	CK ( −)	Negative control
2	CK ( +)	*Burkholderia vietnamiensis* B418 inoculated
3	FOST ( −)	Fosthiazate applied only
4	FOST ( +)	Fosthiazate + *B. vietnamiensis* B418
5	AVM ( −)	Avermectin applied only
6	AVM ( +)	Avermectin + *B. vietnamiensis* B418

### Site preparation

Soil plots (3 m $\times$ 30 m) were treated with the corresponding biopreparations and nematicides to 20 cm depth at the planting holes on February 2, 2018. Transplantation of watermelon seedlings immediately followed treatment implementation. *B. vietnamiensis* B418 was freshly prepared using 100-fold dilutions of laboratory sourced preparations, to a concentration of 5 × 10^8^ CFU/mL. Fosthiazate and avermectin were diluted to 10% and 1.8% w/v respectively as per manufacturers' instructions. Each of the prepared agents was combined with sterilized sand at the ratio 1:5 solution to sand, which was then sprinkled into planting holes at a rate of ca. 50 g/seedling.

### Sampling and soil collection

On April 27, 2018 (Day 85 from transplantation), watermelons were harvested and the samples soil and roots were collected. Rhizosphere soils were extracted from 3 neighboring replicates according to the protocol of Bulgarelli et al^68^. Root systems were sampled from 0-20 cm soil depth and were shaken until approximately 1 mm soil remained attached to the roots. Roots with attached soil were then washed in Falcon tubes filled with PBS buffer (135 mM NaCl, 2.7 mM KCl, 1.5 mM KH_2_PO_4_, and 8 mM K_2_HPO_4_, pH 7.2), followed by shaking at 150 rpm for 30 min. The soil suspension was centrifuged at 8000 rpm for 5 min, and the pellet was collected as rhizosphere soil for analysis. All samples were stored at −80 °C prior to further analysis.

### Assessment of RKN control efficacy

The numbers of nematodes in soil samples and disease severity were determined to evaluate the biocontrol efficacy of different treatments against RKN. The soil samples were processed separately to extract nematode juveniles using Cobb’s sieving and decanting method^69^. Juvenile nematodes were collected and observed under stereomicroscope in a counting dish, with data expressed as juveniles per 100 g of soil.

For the disease severity detection, the gall index (GI) of plant roots were determined on the following scale: 0 = no galling, 1 = 1–25% galling, 2 = 26–50% galling, 3 = 51–75% galling, and 4 = 76–100% galling^70^. The control efficacy was calculated as described by Niu et al^71^. Disease severity, reduction rate and control efficacy were calculated as following formulas:

1 $$\mathrm{Disease\, severity}=\left[\sum \frac{\mathrm{Number\, of\, plants\, with\, root}-\mathrm{knot\, nematode\, disease\, in\, this\, index }\times \mathrm{ Disease\, index}}{\mathrm{Number\, of\, plants\, surveyed }\times \mathrm{ Highest\, root}-\mathrm{knot\, nematode\, disease\, index}}\right]\times 100\%$$

2 $$\mathrm{Reduction\, rate}=\frac{\mathrm{Number\, of\, nematode\, density\, in\, negative\, control\, treatment }-\mathrm{ Number\, of\, nematode\, density\, in\, different\, treatment}}{\mathrm{Number\, of\, nematode\, density\, in\, negative\, control\, treatment}}\times 100\%$$

3 $$\mathrm{Control\, efficacy}=\frac{\mathrm{Disease\, severity\, of\, negative\, control\, treatment }-\mathrm{ Disease\, severity\, of\, different\, treatment}}{\mathrm{Disease\, severity\, of\, negative\, control\, treatment}}\times 100\%$$

### Bacterial and fungal genomic DNA extraction

Genomic DNA was extracted from 0.4 g rhizosphere soil samples using PowerSoil DNA isolation kits (MoBio Labroratories, Carlsbad, CA, USA) according to the manufacturer's instructions. The extracted DNA was electrophoresed on a 1% (w/v) agarose gel, and its mass and concentration were measured using an ultraviolet spectrophotometer (BioSpec-nano, Shimadzu, Japan).

### PCR amplification and amplicon sequencing

The hypervariable V3-V4 region of the bacterial 16S rRNA gene was amplified using the primer pair 341f (5′-CCTACGGGNGGCWGCAG-3′) and 785r (5′-GACTACHVGGGTATCTAATCC-3′)^72^. The internal transcribed spacer-2 (ITS-2) region of fungi was amplified with the primer pair ITS7f (5′-CCAATTTAATCGCAGTGGCTTG-3′) and ITS127r (5′- CGACAGCCGTTTCACAACAATA-3′)^73^. PCR products were sequenced on the Illumina Miseq^TM^ platform with 300 bp length of PE reads by Sangon Biotech (Shanghai) Co., Ltd.

### Real-time quantitative PCR of N-cycling Genes

The abundances of five representative N-cycling genes (*nifH*, archaeal *amoA*, bacterial *amoA*, *nirK*, *nirS*, and *nosZ*) were estimated using standard curves and real-time quantitative PCR on a CFX96 optical real-time detection system (Bio-Rad, Laboratories Inc., Hercules, CA, USA)^74,75^. Each 20 μL reaction contained 10μL of SYBR Green (2X) PCR Master Mix (TaKaRa, Dalian, China), 250 ng μL^-1^ bovine serum albumin (BSA), 0.2μL of the forward and reverse PCR primers (both 20 μM), 1 μL of DNA template (containing 10~20 ng of total DNA) and 8.6 μL of double-distilled water (ddH_2_O). The primer sequences and references used in qPCR are listed in Table 4. Standard curves were obtained using three replicates of serial dilutions of linearized plasmids containing fragments of *nifH*, archaeal *amoA*, bacterial *amoA*, *nirK*, *nirS*, or *nosZ* genes. The specificity of the qPCR procedure was determined by melting curve analysis and agarose gel electrophoresis.

**Table 4:** N-cycling genes primers used for qPCR quantification assays.

Genes	N-cycling functions	Primers	Primer sequences
*nifH*	N fixation	PolF PolR	5′-TGCGAYCCSAARGCBGACTC-3′ 5′-ATSGCCATCATYTCRCCGGA-3′
Archaeal *amoA*	Archaeal ammonia oxidation	CrenamoA23f. CrenamoA616r	5′-ATGGTCTGGCTWAGACG-3′ 5′-GCCATCCATCTGTATGTCCA-3′
Bacterial *amoA*	Bacterial ammonia oxidation	amoA-1F amoA-2R	5′-GGGGTTTCTACTGGTGGT-3′ 5′-CCCCTCKGSAAAGCCTTCTTC-3′
*nirK*	Cu-nitrite reductase	nirK876 nirK1040	5′-ATYGGCGGVAYGGCGA-3′ 5′-GCCTCGATCAGRTTRTGGTT-3′
*nirS*	Cd-nitrite reductase	nirSCd3aF irSR3cd	5′-AACGYSAAGGARACSGG-3′ 5′-GASTTCGGRTGSGTCTTSAYGAA-3′
*nosZ*	Nitrous oxide reductase	nosZ1F nosZ1R	5′-WCSYTGTTCMTCGACAGCCAG-3′ 5′-ATGTCGATCARCTGVKCRTTYTC-3′

### Bioinformatics analysis

The bioinformatic analysis was performed by removing primer adapter sequences with cutadapt (vs1.2.1), removing low-quality bases (Phred Quality Score = 20), and splicing, discarding low-quality sequences shorter than 100 bp in length (≈10% of the total sequences), and removing the specific amplification sequences and chimeras with Uchime (vs4.2.40) to obtain the effective value of sequence data for each sample^76^. Afterwards, clean reads of bacterial 16S and fungal ITS rRNA sequences were divided into operational taxonomic units (OTUs) at 97% similarity using QIIME (vs1.8.0) with the UCLUST algorithm^77^. Annotation of the representative sequences of OTUs was performed by BLAST (vs2.2.30) against the Silva database (release 123) with similarity > 90% and coverage > 90%^78^.

### Statistical analysis

The Vegan software package (vs3.4.3, Team 2013) in R was used for statistical analysis. The variance stabilizing transformation method in DESeq2 package (vs1.18.1) was applied to normalize the library sizes between samples^79^. Analysis of similarity between microbial communities was conducted by principal component analysis (PCA) according to weighted UniFrac distances matrix between sample OTUs composition using the Phyloseq package and Vegan package (vs2.4-5)^80^. Constrained principal component analysis was performed using the same weighted UniFrac distances by capscale () function in the Vegan package to test the effects on OTU composition of two experimental factors of B418 treatment and other application of nematicides and biopreparations. OTU composition was also analyzed using Bray–Curtis distance.

The alpha diversity measured by Shannon index was estimated after rarefying the number of sequences in each sample according to the sample with the minimum number of sequences, using the Vegan package. Relative abundances of predicted microbial functions between experimental treatments were compared using the Statistical Analysis of Metagenomic Profiles (STAMP) package (vs2.1.3)^81^.

## Data Availability

Raw sequence data reported in this paper are available in Genome Sequence Archive (GSA; http://bigd.big.ac.cn/gsa) in BIG Data Center, Chinese Academy of Sciences under the accession number CRA006248.
